# Africa’s booming rice cultivation is fueling regional warming

**DOI:** 10.1038/s41598-025-27436-5

**Published:** 2025-12-01

**Authors:** Basudev Swain, Marco Vountas, Aishwarya Singh, Rui Song, Nidhi L. Anchan, Nisha Patel, Debashis Tripathy, Biswa R. Swain, Dukhishyam Mallick, Richard Alawode, Sachin S. Gunthe

**Affiliations:** 1 Atmospheric, Oceanic and Planetary Physics, University of Oxford, Oxford, UK; 2 Institute of Environmental Physics, University of Bremen, Bremen, Germany; 3 Centre for Atmospheric and Climate Sciences, Indian Institute of Technology Madras, Chennai, India; 4 School of Earth and Atmospheric Sciences, Georgia Institute of Technology, Atlanta, GA USA; 5 Biogeochemistry Department, Max Planck Institute for Chemistry, Mainz, Germany; 6 National Centre for Earth Observation, Atmospheric, Oceanic and Planetary Physics, University of Oxford, Oxford, UK; 7 Department of Energy, Environmental, and Chemical Engineering, Washington University, Saint Louis, MO USA; 8 Department of Environmental Meteorology, University of Kassel, Kassel, Germany; 9 Yusuf Hamied Department of Chemistry, University of Cambridge, Cambridge, UK; 10 Chester F. Carlson Center for Imaging Science, Rochester Institute of Technology, Henrietta, USA; 11 IJCLab Orsay, CNRS/IN2P3, Université Paris-Saclay, Saclay, France; 12 Leipzig Institute for Meteorology, Leipzig University, Leipzig, Germany

**Keywords:** Increasing surface air temperature, Greenhouse gases, Rice cultivation, Climate sciences, Climate change, Climate-change impacts, Environmental health

## Abstract

The significant increase of surface air temperature in Africa during the recent industrial period has been previously attributed to emissions from rapidly growing urbanization and industrial emissions. This study highlights the rapid growth of rice cultivation as another major influencing factor. We estimate that a 436% (14 million hectares) surge in rice cultivation area during the industrial period (1960-2018) in the sub-Saharan African region is associated with an increase of 603 million tons of agricultural methane emissions, making it the largest source of methane among all sectoral contributors, including energy, industrial processes, waste, land-use change, and forestry. These changes are further associated with an increase in the total surface air temperature anomaly to 1.3$^\circ$C, with greenhouse gas (GHG) forcing alone accounting for a rise from 0.47$^\circ$C to 0.92$^\circ$C throughout the industrial era compared to pre-industrial baseline (1850–1900), as estimated using the Regular Optimal Fingerprinting (ROF) method. Continued rice cultivation expansion to feed Africa’s rapidly growing population holds the potential for further intensifying current and future warming conditions. However, adopting more sustainable rice farming practices can help to reduce emissions and mitigate these effects.

## Introduction

Africa’s population now exceeds 1.4 billion, making it the second most populous continent^1^. This rapid growth presents various challenges, including increased pressure on food demand^2^. Traditionally, African diets have relied on staple crops such as maize, sorghum, and cassava^2^. However, the rising population over the last few decades has marked a shift towards rice as a staple food^2–4^. To address the recent global food price crisis, a coalition of sub-Saharan African countries was formed to boost rice production across 23 nations of the region^5^. Africa possesses a calculated 20 million hectares of unused land appropriate for agricultural rice farming^5^, together with ample unexploited resources of water beyond just rainfall for agricultural purposes^6^. The initial plan aimed to increase rice production twice, while the second phase targets doubling it again to attain self-sufficiency in rice as a staple food by 2030, expanding to include nine additional countries^5^. However, the rapid increase in rice cultivation has significant climate implications due to the emission of methane ($CH_4$), a potent greenhouse gas^7–10^. Methane, which has contributed approximately 0.6$^\circ$C to global warming since the pre-industrial period (1850-1900)^11^, is released from various human activities such as the mining of coal, the management of livestock, proper waste dumping, and farming of rice^7,11^.

Rice cultivation, particularly under flooded (anaerobic) conditions, is one of the most significant anthropogenic sources of methane globally^12,13^. When rice paddies are inundated, the oxygen-deprived environment creates ideal conditions for methanogenic archaea to break down organic matter in the soil, resulting in substantial methane release to the atmosphere^13–15^. The methane escapes through diffusion, ebullition, and plant-mediated transport via the aerenchyma tissue in rice plants^13–16^. This mechanism has been thoroughly examined in previous studies^13–16^. Africa’s rice cultivation accounts for approximately 7% of total anthropogenic global methane release and represents the largest source of agricultural methane^7^. In tropical and subtropical areas, mostly the parts of Africa where rice cultivation has expanded rapidly since the 1960 s due to food security needs, this sector has became a dominant source, especially in lowland, irrigated, or rainfed-flooded systems where anaerobic conditions are sustained^7,17–20^.

It is noteworthy that while recent valuable studies highlights that rice production is a source of methane emissions^7,20^, to the best of our knowledge, no research has yet been conducted to explore the association between methane emissions from the rapid expansion of African rice cultivation area and its impact on rising regional surface air temperature (SAT) or regional climate change, despite evidence of substantial methane emissions from this agricultural activity. Thus, we are the first to link methane emissions from rice cultivation in Africa with regional SAT increases.

The rise in SAT over Africa in the recent industrial period (1960-2018) has become increasingly pronounced^21,22^, leading to more frequent heatwaves affecting vulnerable ecosystems and populations^23,24^. Previously, it was thought that the rise in SAT in Africa during the recent industrial period was due to emissions from rapid urbanization, industrialization^25–27^. Therefore, it is important to assess the rise in SAT during the industrial period (1960–2018) relative to the pre-industrial period (1850–1900).

Here, we demonstrate that the rapid increase in rice cultivation is another significant contributor to the accelerating rise in SAT in Africa. Specifically, we now highlight how the Coalition for African Rice Development (CARD)^3,28^ effort with the the ambition to increase rice yields two-fold in the countries of sub-Saharan African region^3,28,29^, contributed to a significant expansion in rice cultivation area. While this development has addressed regional food security concerns, it has also inadvertently intensified methane emissions due to increased paddy field area, a key driver of methane-driven warming in the region. By drawing this connection, we aim to clarify the dual challenge of balancing agricultural productivity with climate mitigation, thereby reinforcing the relevance and urgency of our analysis. Further, by adopting a sustainable approach to rice cultivation, rather than relying on traditional methods^30–32^, to feed Africa’s rapidly growing population has the potential to minimize the intensification of current as well as future heat conditions.

## Results

### Rising SAT in Africa during the industrial period

**Fig. 1:**
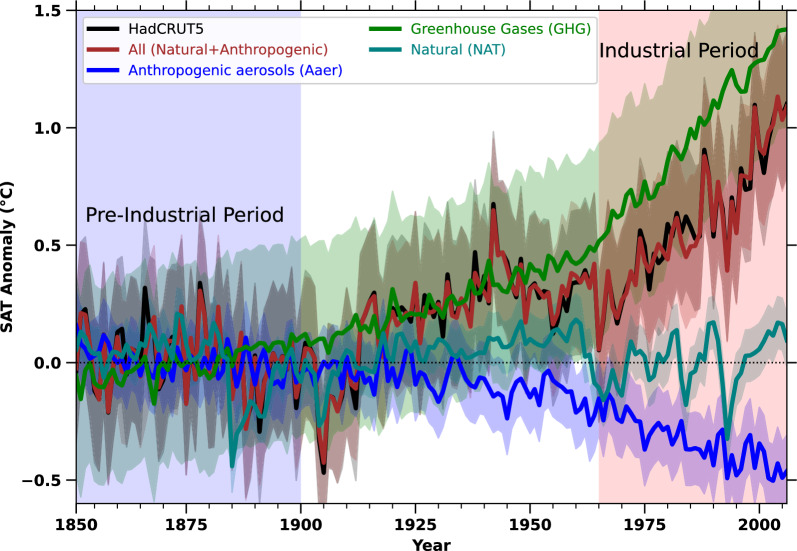
The annual anomaly of GHG, Aaer, and NAT Surface Air Temperature in Africa during the pre-industrial and industrial periods. The figure shows the annual SAT anomaly of different forcings in Africa. The anomaly is calculated with respect to the pre-industrial period from 1850 to 1900. The forcing ALL= Natural+Anthropogenic forcings. The Natural forcings considered solar insolation and volcanic eruptions. The shaded line shows a confidence interval of 95%. The pre-industrial and industrial periods are shown as blue and red shades.

To select CMIP5^33^ models for further analysis (listed in Table S1), we used HadCRUT5 observational SAT data^34^ as a reference. The HadCRUT5 SAT dataset is considered due to its long-term consistency, robust observational basis, and wide use in climate studies^34^. HadCRUT5 combines land (CRUTEM5) and ocean (HadSST4) temperature records and includes uncertainty estimates, which is particularly valuable for data-scarce regions like Africa^34,35^. Unlike reanalysis products, which rely on model assimilation that can introduce temporal inhomogeneities, HadCRUT5 offers a more stable record for evaluating long-term trends from the pre-industrial era to the present^34^. Its widespread use in CMIP model evaluation and IPCC assessments also ensures consistency with our modeling framework^8,36–38^. Only models with a correlation coefficient (R) of $\ge$ 0.5 over the African continent during the industrial period were included (see Methods section, Fig. S1). The selected models are NorESM1-M, MPI-ESM-LR, IPSL-CM5A-LR, CCSM4, IPSL-CM5A-MR, GFDL-CM3, MIROC5, CSIRO-Mk3-6-0, HadGEM2-CC, CNRM-CM5, GISS-E2-H, FGOALS-g2, HadGEM2-ES, MIROC-ESM, GISS-E2-R, CanESM2, Bcc-csm1-1, and BNU-ESM. These models were used to quantify the impact of greenhouse gas (GHG), aerosols (Aaer), natural forcings (NAT) on SAT changes over Africa from the year 1850 to the year 2005 (Fig. 1). Annual SAT anomalies due to these forcings were calculated by subtracting the average SAT of the pre-industrial period (1850–1900) from that of the historical period (1850–2005). Fig. 1 reveals a positive SAT anomaly due to all forcings (All = Anthropogenic + Natural) starting from 1915, with a transition from negative to positive anomalies. A steep rise in SAT anomalies is observed from the onset of the industrial period (1965 onwards), aligning closely with the SAT anomalies in the HadCRUT5 observations.

To understand the drivers behind the transition from negative to positive SAT anomalies after 1915 and the steep rise in SAT from 1965 onwards, it is crucial to analyze the individual contributions of GHG, Aaer, and NAT forcings. Fig. 1 clearly shows that GHG forcings have led to a substantial increase in SAT starting from 1915, with an even sharper rise after 1965, marking the onset of the industrial period. In contrast, anthropogenic aerosols had minimal impact before 1935 but contributed significantly to cooling during the industrial period (from 1965 onwards), likely due to increased aerosol emissions from industrial activities in Africa^39,40^. This cooling effect is attributed to the higher aerosol load, which absorbs and scatters incoming solar radiation and creates a dimming effect^41–47^. The influence of natural forcings (NAT) on the overall SAT rise over Africa is negligible (Fig. 1). This highlights that the regional warming over Africa during the industrial period, with an anomaly of approximately 1.4$^\circ$C, is predominantly caused by greenhouse gas emissions (Fig. 1). Moreover, Fig. 1 reveals that the warming (positive anomaly) due to GHG forcings exhibits a steep increase, exceeding the SAT rise observed in the HadCRUT5 dataset.

In addition to analyzing the contributions of individual forcings to the overall warming (rise in SAT anomaly) over the African region, this study makes use of Regular Optimal Fingerprinting (ROF) method^48^ to further quantify the roles of GHG, Aaer, and NAT forcings to the HadCRUT5 SAT and multi-model-mean SAT (see Methods section). ROF is a powerful statistical tool that offers significant advantages in isolating and quantifying the specific effects of various climate drivers–such as greenhouse gases, aerosols, and natural variability–on SAT changes. Unlike anomaly-based analysis, which identifies deviations from a baseline, ROF enables precise attribution of SAT changes to individual forcings, thereby providing insights into their relative contributions. This approach not only complements anomaly analysis but also enhances climate assessments by offering detailed causal explanations for temperature variations. Such complementary insights are crucial for understanding regional and global SAT trends and the ROF method has been successfully utilized in several key studies^8,48–52^ to detect and attribute the influence of specific forcings on SAT rise.

In this study, the ROF method is used to assess the impact of individual forcings on the overall rise in SAT over Africa^48^. To complement the SAT anomaly analysis, which primarily compares modeled responses under GHG forcings with observed warming patterns, we considered the ROF framework to formally determine the factors of individual climate forcings to total surface air temperature anomaly over Africa. While anomaly analysis illustrates broad trends, it does not quantify the influence of specific forcings^48^. The ROF method provides a statistical framework to assess the degree to which observed SAT trends in the HadCRUT5 dataset can be associated with GHGs, aerosols, and natural factors, offering a more rigorous attribution approach^8,48^. This allows us to isolate the dominant drivers of regional warming and validate whether modeled fingerprints of forcing align with observed changes^8,48–52^. The ROF method estimates that between 0.47$^\circ$C to 0.92$^\circ$C (5–95% range) of the total SAT increase in Africa is attributed to greenhouse gas forcings. In contrast, anthropogenic aerosols contribute a cooling effect ranging from −1.82$^\circ$C to −1.36$^\circ$C, while natural forcings have a negligible impact, ranging from −0.03$^\circ$C to 0.06$^\circ$C. These results highlight that the primary driver of SAT rise in Africa is greenhouse gas forcing. Additionally, we investigate the relationship between this rapid SAT increase during the industrial period (1960–2018) and the rise in rice cultivation, associated methane emissions, and GHG forcings in the following section.

### Correlation between rice cultivation, agricultural methane emissions, and SAT anomalies in Africa

Understanding the interconnected relationship, such as, time-series comparison, and regression analysis between rice cultivation area, agricultural methane emissions, and surface air temperature anomalies is essential to assess the potential role of increased rice farming in regional warming over Africa during the industrial period. In this section, we examine these relationships using methane emissions from the broader agricultural sector and total SAT across Africa, acknowledging that rice cultivation is a significant contributor to agricultural methane emissions in the region^7,20^.

Despite Africa experiencing pronounced and multifaceted climate change impacts, the continent faces a severe lack of ground-based observational data and focused scientific studies. This data gap hinders efforts to accurately isolate and attribute the contribution of rice cultivation–driven emissions to regional warming. Given these limitations, we focus on establishing correlations among variables rather than asserting direct causality.

**Fig. 2:**
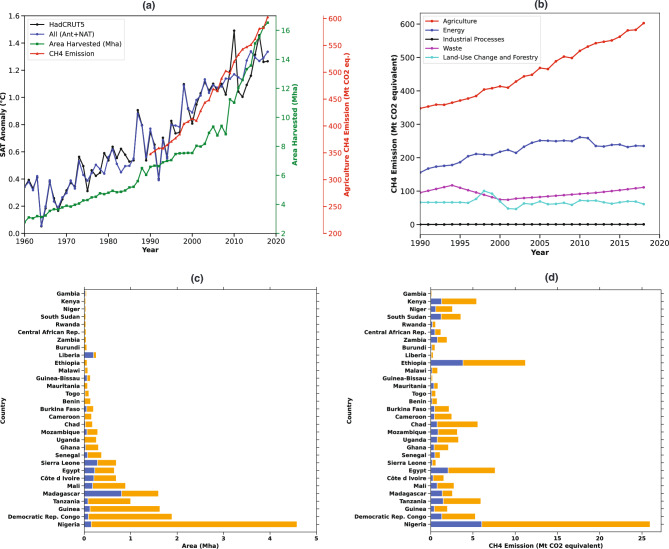
The temporal relationship between increased rice production, as measured by area harvested, and its impact on methane emissions and surface air temperature changes across Africa during the recent Industrial period (1960-2018). a) Time-series data for the recent industrial period showing area harvested for rice cultivation, associated agricultural methane emissions, and the anomaly is calculated with respect to the pre-industrial period from 1850 to 1900. b) Methane emissions from various sectors, such as Agriculture, Energy, Industrial processes, Waste, and Land use change. c) and d) Bar plots depicting changes in harvested area and methane emissions, respectively. Blue indicates harvested area and emissions in the beginning year (1960) of the industrial period, while orange shows the increase in area harvested and methane emissions from 1960 to 2018.

Our analysis demonstrates a consistent and pronounced temporal increase in all three key variables–area harvested for rice cultivation, agricultural methane emissions, and surface air temperature anomalies throughout the industrial era (Fig. 2a). Notably, the agricultural sector emerges as the largest contributor to methane emissions across Africa, surpassing other major sectors such as energy, industrial processes, waste, and land-use change and forestry (Fig. 2b). The area harvested for rice cultivation has shown a consistent and steep increase from 1960 to 2018 (Fig. 2a). The rise in rice production has primarily been achieved through the expansion of cultivated areas primarily in sub-Saharan Africa (SSA) countries driven by CARD initiative^3,28,29^ to meet the growing demand (Fig. 2c). This indicates that the area for rice cultivation has grown approximately 436% in 2018 compared to 1960 over Africa (Fig. 2c), expanding to 14 million hectares. In contrast, China, the world’s largest rice producer, farms rice over a land coverage totaling 30 million hectares^53^. Most of the enlargement in rice cultivation has occurred in West African nations (Fig. 2c,d). Sub-Saharan areas of Africa have significantly enhanced agricultural production by using fertilizers and enhancing irrigation^3^. Effective water management and proper irrigation projects funded by the World Bank have facilitated the development of extensive irrigated rice fields by tapping into previously unused water resources^54^. This irrigation often enables rice cultivation two times a year. Additionally, the application of high-content nitrogen associated fertilizers has been employed to boost rice production^3,28,29^. Alongside the expansion of rice cultivation, agricultural methane emissions have experienced a significant increase during the same period (Fig. 2c,d). Methane is a highly potent greenhouse gas with a much greater global warming potential compared to carbon dioxide^55^. Overall, methane emissions have surged from 347 million tons in 1990 to 603 million tons in 2018 (Fig. 2a). Given that rice cultivation is a major methane source^7,20^, this rise aligns with the broader warming trends over Africa. A latest global ground-based measurement study^56^ on emissions from rice production has examined various agricultural practices together with environmental conditions, such as various textures of the soil, techniques of rice planting, and seasonal water availability for irrigated fields. Unlike aerobic rice cultivation, which is farmed in well-aerated soils and does not produce methane emissions^57^, rainfed lowland, irrigated, and deepwater rice cultivation are the primary sources of methane emissions^57^. Methane is produced in flooded rice fields where oxygen-deprived soils facilitate methanogenesis by archaeavcouwenberg2012towards,dubey2005microbial. Methane escapes via diffusion, ebullition, and through aerenchyma tissues in rice plants^13^.

Whereas, upland rice is typically grown in elevated tropical regions with good inherent drainage, rain-dependent areas that do not collect runoff water, often lacking aquifer-fed water distribution, generally without bunds, on areas with slopes ranging from zero to greater than thirty percent^57,60,61^. However, the proportion of upland rice farming in Africa has declined in recent decades (2000-2020), averaging 30% during the industrial period^57,62^. In Nigeria, the International Rice Research Institute^4^ initially designated 51% of the rice area for upland cultivation, but the more recent Global Upland Area database for the industrial period reports only 29%^57^. This shift has a significant impact on methane emissions (Fig. 2c, d). Nigeria, the largest contributor to increased rice cultivation, expanded its harvested area from 0.14 Mha in 1960 to 4.5 Mha in 2018 (Fig. 2c). Consequently, methane emissions from the combined agricultural activities in Nigeria rose from 6 million tons in 1960 to 26 million tons in 2018 (Fig. 2d).

**Fig. 3:**
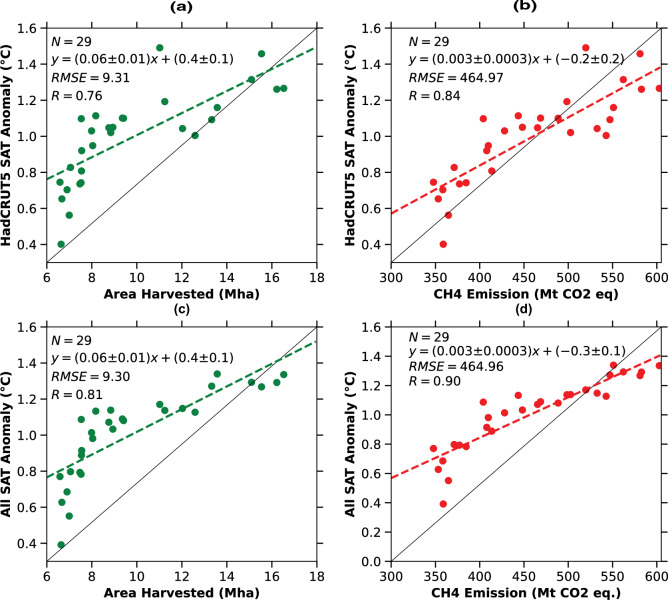
The correlation between increased rice production, as measured by area harvested, and its impact on methane emissions and surface air temperature changes across Africa during the recent Industrial period (1960-2018). a-d) Linear regression (correlation) analysis between surface air temperature anomalies observed by HadCRUT5 and simulated by multi-model means (All) with the area harvested and methane emissions during the industrial period, respectively.

Further, investigation of the rise in surface air temperature anomalies, a key indicator of African climate change, has similarly exhibited a steep upward trend (Fig. 2a). From 1960 to 2018, the surface air temperature anomaly over Africa has increased from an average of 0.3$^\circ$C relative to the pre-industrial period baseline to 1.3$^\circ$C in both HadCRUT5 observational data as well as multi-model-mean simulations (Fig. 2a). This rise in temperature anomalies is a direct consequence of increased global and regional greenhouse gas emissions in the atmosphere, contributing to the enhanced greenhouse effect on Africa warming (Fig. 1). However, the accelerated expansion of rice cultivation and the associated increase in methane emissions may be further fueling the regional warming trends over Africa. The correlation of the current increase in rice cultivation, methane emissions on the rise in SAT anomalies, underscores a significant linear interrelationship (Fig. 3a-d). The expansion of rice cultivation directly contributes to higher agricultural methane emissions, which in turn fuels the regional greenhouse effect and accelerates the warming over Africa, as it can be seen in the (Fig. 3a-d, and Fig. 1). Further, the comparison between the HadCRUT5 observations (Fig. 3a) and multi-model-mean (All SAT) (Fig. 3c) surface air temperature anomaly with the area harvested, and methane emissions (Fig. 3b,d) during the industrial period shows a very strong Pearson correlation coefficient (R) of greater than 0.90 (Fig. 3a-d). The strong temporal alignment and high Pearson correlation coefficients among rice cultivation, agricultural methane emissions, and SAT anomalies suggest a potential linkage, providing foundational evidence to support future research. These correlations emphasize the pressing necessity for sustainable farming methods that can curb methane emissions while maintaining food security across Africa^30–32^. The rise in methane emissions associated with agricultural activities is further corroborated by the sectoral emission time series shown in Fig. 2b.

While we acknowledge that these correlations do not imply causation, this study establishes a critical stepping stone for future studies. Advanced regional climate modeling, combined with high-resolution, multi-source satellite observations, will be essential to unravel the complex causal interactions between rice cultivation, methane emissions, and regional warming over Africa.

### Spatial SAT trends in various forcings and their contributions over Africa

**Fig. 4:**
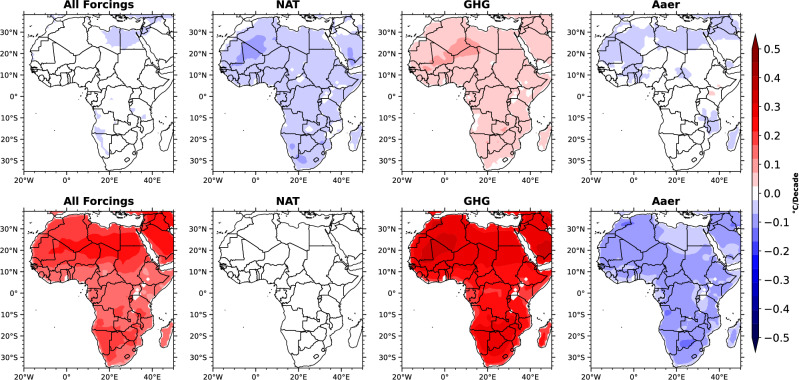
Spatial map of surface air temperature trends ($^\circ$C/Decade) due to combination of All forcings, Natural (NAT), Greenhouse gases (GHG), and Anthrogoneic aerosol (Aaer) forcings during the pre-industrial (top row) and industrial period (bottom row) respectively. The top and bottom panel shows the SAT trend for the pre-industrial and industrial periods for All forcings, NAT, GHG, and Aaer respectively.

To evaluate the spatially resolved contributions of various climate forcings to surface air temperature (SAT) trends over Africa, we analyzed the individual impacts of well-mixed greenhouse gases (GHGs), anthropogenic aerosols (Aaer), and natural (NAT) forcings using CMIP5 multi-model single-forcing simulations across during both the early pre-industrial and later industrial eras (Fig. 4). Our results reveal a clear intensification of GHG-driven warming, with SAT trends increasing from approximately 0.04$^\circ$C per decade during the pre-industrial period to 0.26$^\circ$C per decade in the industrial era. This substantial rise underscores the dominant role of global and regional GHGs emissions in driving regional warming over Africa.

In contrast, Aaer forcings exhibit enhanced cooling over the same timeframe, with trends shifting from −0.03$^\circ$C/decade to −0.06$^\circ$C/decade (Fig. 4). This cooling effect is attributed to both the direct radiative effects of anthropogenic aerosols, which scatter incoming solar radiation and reduce the net energy reaching the surface, and their indirect effects on cloud microphysics, which increase cloud albedo and lifetime, further enhancing atmospheric reflectivity^41–47^. These mechanisms collectively contribute to a net negative radiative forcing that offsets part of the GHG-induced warming.

Natural (NAT) forcings, comprising solar variability and volcanic aerosols, play a relatively minor role, with associated SAT trends changing from −0.04$^\circ$C/decade to approximately 0$^\circ$C/decade, indicating a near-neutral effect in the industrial period. These results emphasize that the warming observed over Africa is primarily driven by anthropogenic GHGs, while aerosols exert a moderating influence, and natural variability contributes minimally in recent decades (Fig. 4).

It is important to note that SAT responses to GHG forcings in global climate models arise from globally well-mixed gases, whose radiative forcing is spatially uniform and not limited to their geographic source regions. Therefore, the warming simulated over Africa reflects global emissions. However, regionally emitted GHGs–such as methane from Africa’s expanding rice cultivation–can fuels disproportionately to local radiative forcing and warming. This highlights the critical need for future high-resolution observational and modeling studies to quantify the regional contributions of specific emission sectors to SAT changes across the African continent.

## Discussions

Understanding the interconnected links between agricultural practices, greenhouse gas emissions, and climate change in Africa is crucial for devising sustainable solutions. During the industrial period, the area dedicated to rice cultivation has increased by approximately 436% (14 Mha), which is associated with a rise of 603 million tons in agricultural methane emissions. It is important to clarify that the observed rise of 603 billion tons in agricultural methane emissions over the industrial period (1955–2018) reflects emissions from the entire agricultural sector, not solely from rice cultivation (Figs. 2a-d, 3a-d). However, rice cultivation is a well-documented source of methane due to the anaerobic conditions of flooded paddy fields, and the significant increase in rice harvested area across Africa during this period suggests a likely contribution. While exact attribution is limited by data availability, the parallel trends in rice expansion and agricultural methane emissions indicate a strong correlation, which we explore in this study as a foundational step toward future causal assessments. These agricultural methane changes are linked to an increase in the total temperature anomaly to 1.3$^\circ$C, with greenhouse gas (GHG) forcing alone contributing to an increase in temperature from 0.47$^\circ$C to 0.92$^\circ$C relative to the pre-industrial period.

Methane $(CH_4)$ emissions from rice cultivation play a critical role in amplifying regional and global warming due to their strong radiative forcing characteristics. Unlike carbon dioxide, methane exhibits a markedly higher capacity to trap heat in the atmosphere approximately 28 to 34 times greater over a 100-year time horizon, making it one of the most potent greenhouse gases in the atmosphere^63,64^. In flooded rice paddies, anaerobic soil conditions foster methanogenic microbial activity^13–15^, which leads to sustained methane production and release throughout the growing season. Once emitted, methane not only contributes directly to warming by absorbing outgoing longwave radiation^63,64^ but also exerts indirect effects through its role in tropospheric ozone formation and interference with hydroxyl radicals, which regulate the atmospheric lifetimes of other gases^63,64^. The combination of these direct and indirect pathways makes rice-related methane emissions a critical driver of radiative forcing, particularly in regions where rice cultivation has expanded rapidly^7,63,64^. Understanding these mechanisms is essential for attributing agricultural contributions to observed surface air temperature (SAT) trends and for informing targeted climate mitigation strategies.

The steep upward trends in harvested rice area, methane emissions, and surface air temperature anomalies during the industrial period highlight the interrelated effects of agricultural activities on rising temperatures across Africa (Fig. 2a-d). This, in turn, affects its fragile ecosystem and rapid climate change. Addressing these issues requires integrated approaches that consider the impacts of agricultural expansion on greenhouse gas emissions and local temperature rise. Sustainable practices, technological innovations, and effective policy measures are essential to mitigate these impacts and promote environmental and climatic sustainability. Meeting the food needs of Africa’s rapidly increasing population will likely lead to further growth and intensification of rice farming in the near future. This situation underscores the need for enhanced efforts to curtail methane emissions, while also addressing the rising surface air temperatures and their significant effects on Africa’s rapidly evolving climate.

The observed relationship between rice cultivation expansion (Figs. 2a-d, 3a-d) methane emissions, and surface air temperature anomalies in sub-Saharan Africa highlights the need for targeted climate-smart agricultural strategies. Given the growing importance of rice as a staple food and the rapid intensification of its cultivation, incorporating low-emission practices such as alternate wetting and drying (AWD), improved irrigation scheduling, and the use of methane-inhibiting rice varieties can significantly curb methane emissions without sacrificing yield^30–32^. These findings also underscore the importance of aligning agricultural development programs, such as the Coalition for African Rice Development (CARD)^3,28^, with climate mitigation efforts. By embedding such evidence into national adaptation plans and regional policies, stakeholders can promote sustainable intensification that supports food security while minimizing climate impacts. This study thus provides a timely contribution to guiding both agricultural innovation and climate policy in Africa.

## Data and methods

### SAT observation, model simulations

This study examined 22 climate models and 158 corresponding simulations, spanning the 19th, 20th, and 21 st centuries, as summarized in Table S1. It is noteworthy that every type of forcings considered in this study is not available in every model run. The analyzed models cover a variety of emission scenarios, including RCP4.5, and incorporate different forcings: 17 simulations account for natural (NAT) forcings (solar and volcanic), 18 include greenhouse gas (GHG) forcings, 10 represent anthropogenic aerosol (Aaer) forcings, and 5 incorporate land-use (LU) forcings. These experiments, which contribute significantly to the IPCC Fifth Assessment Report^65^, aim to replicate climate variability and changes across the 19th, 20th, and 21 st centuries by accounting for diverse forcings. Monthly mean surface air temperature (SAT) data were obtained from the 22 CMIP5 models for the RCP4.5 scenario and historical simulations. For the post-2005 period, the RCP4.5 scenario was used to extend the analysis through 2018, as it provides a more realistic representation of current climate conditions. This scenario represents an intermediate greenhouse gas (GHG) stabilization pathway, assuming the implementation of climate policies that lead to radiative forcing levels stabilizing at 4.5 W/m² by 2100. RCP4.5 is widely used in climate attribution and impact studies due to its policy-relevance and balanced emission trajectory^66,67^, reflecting neither the most extreme (e.g., RCP8.5) nor the most conservative (e.g., RCP2.6) future scenarios. Moreover, RCP4.5 offers broad model participation and compatibility with the CMIP5 historical simulations used in our analysis, allowing for seamless transition from observed to projected periods and ensuring consistency in the comparison of anthropogenic influences on surface air temperature anomalies^66,67^.

To evaluate the models’ performance, Taylor diagrams have been used to compare the mean SATs model estimated by CMIP5 models with HadCRUT5 observed SAT data^34^ over Africa from 1955 to 2005. This timeline was selected because of the higher-quality observational data from satellite measurements. Models with correlation coefficients $\le$ 0.5 relative to the HadCRUT measurements are removed, thus only 15 different models are considered, such as NorESM1-M, MPI-ESM-LR, IPSL-CM5A-LR, CCSM4, IPSL-CM5A-MR, GFDL-CM3, MIROC5, CSIRO-Mk3-6-0, HadGEM2-CC, CNRM-CM5, GISS-E2-H, FGOALS-g2, HadGEM2-ES, MIROC-ESM, GISS-E2-R, CanESM2, Bcc-csm1-1, and BNU-ESM. Taylor diagram shown in Fig. S1 shows the performance of CMIP5 model simulations against observational data. Specifically, we note that CMIP5 models often rely on simplified representations of land-use change and may not fully resolve fine-scale processes such as water management practices, soil conditions, or region-specific methane emissions from rice paddies. Similarly, while HadCRUT5^34^ provides a comprehensive observational temperature dataset, it does not directly account for land-use-specific surface feedbacks, such as albedo changes or biogeochemical fluxes, that are especially relevant to paddy agriculture. We believe that acknowledging these limitations strengthens the transparency and robustness of our methodological framework and encourages future work that incorporates higher-resolution or region-specific land-use and agricultural datasets.

Further, to ensure methodological consistency and transparency, we accounted for differences in forcing availability across the CMIP5 models. Specifically, while GHG, Aaer, and Nat forcing experiments were available across a broader range of models, the land-use (LU) forcing experiments were limited to outputs from five models. Accordingly, analyses involving LU forcings were not conducted using only this subset to maintain consistency and avoid mixing across unequal ensembles. For each forcing, the multi-model mean was computed independently, and inter-model variability was represented by ±1 standard deviation. This approach allows for a balanced comparison of forcing-specific effects while transparently capturing associated uncertainties. Details on the number of models used for each experiment are provided in Table S1.

To guide readers more effectively, we have included a brief summary of Table S1. The table lists the 22 CMIP5^33^ climate models used in our study, detailing their availability of historical simulations and the specific forcings included. Among these models, all provide historical simulations, with 18 including well-mixed GHG forcings, 10 incorporating Aaer, and 17 featuring NAT forcings. This diversity of model-forcing combinations allows us to robustly assess the individual and combined impacts of different anthropogenic and natural drivers on surface air temperature trends.

Observed SAT is calculated as an annual anomaly using area-weighted averages for the African continent from the HadCRUT5 dataset, with anomalies calculated relative to the pre-industrial baseline (1850–1900). The median dataset was used to analyze results and reduce uncertainties. To ensure consistency across datasets, SAT data from different models and simulations were re-gridded to a 1$^\circ$ $\times$ 1$^\circ$ resolution using the Climate Data Operators (CDO) toolkit^68^. Trend estimation employed the Iteratively Reweighted Least Squares (IRLS) method^69^.

Further, in this study, the countries are selected (Fig. S2) on the basis of the Coalition for African Rice Development (CARD)^3,28,29^ has significantly influenced rice cultivation strategies across the continent. Through its investment frameworks and national agricultural plans, CARD encourages African countries to boost agricultural productivity, and to improve food security. In this context, many African nations have prioritized rice as a key strategic crop due to its growing importance in food systems and rising demand. This policy emphasis has contributed to the steady increase in rice harvested area across several regions, particularly in West Africa, aligning with broader CARD goals of agricultural transformation and economic development.

### Regular Optimal Fingerprinting (ROF) method

Additionally, a ROF method analysis was performed^48^ by using HadCRUT5 observations, and different CMIP5 model runs, implemented in Python^70^. This approach evaluates the influence of individual forcings to the total SAT change observed by HadCRUT5 to SAT fluctuations^49^. The ROF method has been successfully utilized in several key studies^8,48–52^ to detect and attribute the influence of specific forcings on SAT rise. The calculation process of ROF steps are outlined below:

The step begins by modeling by using HadCRUT observed temperature change ($y$) as regression of various model simulated forcing (such as GHG, Aaer, NAT) ($x_1, x_2, \ldots , x_n$), and ($\epsilon$, which is residual value):

$$\begin{aligned} y = \beta _0 + \beta _1 x_1 + \beta _2 x_2 + \ldots + \beta _n x_n + \epsilon \end{aligned}$$

Then the SAT values are arranged as matrix for computational efficiency. $X$ represents the different considered forcings matrix, $Y$ is the observed SAT vector, and $\beta$ the array of coefficients to be inferred:

$$X = \begin{bmatrix} 1 & x_{1,1} & x_{2,1} & \ldots & x_{n,1} \\ 1 & x_{1,2} & x_{2,2} & \ldots & x_{n,2} \\ \vdots & \vdots & \vdots & \ddots & \vdots \\ 1 & x_{1,m} & x_{2,m} & \ldots & x_{n,m} \end{bmatrix}$$

$$Y = \begin{bmatrix} y_1 \\ y_2 \\ \vdots \\ y_m \end{bmatrix}$$

$$\beta = \begin{bmatrix} \beta _0 \\ \beta _1 \\ \vdots \\ \beta _n \end{bmatrix}$$

Thus, the regression can be written as $Y = X \beta + \epsilon$.

Then to calculate the coefficient associated with ($\beta$) we apply least squares approach to reduce the summation of the exponent of 2 residuals Within HadCRUT observation and predicted SAT: $[ \beta = (X^T X)^{-1} X^T Y ]$

In addition, we compute remaining discrepancy ($\epsilon$), these are the disparity within HadCRUT5 SAT and predicted SAT. The model uncertainties are considered as the standard deviation of the obtained residuals ($STD\_residual$).

Next, estimation of confidence intervals (CI) at 95% of the ($\beta _i$) are computed as:

$$\begin{aligned} \text {CI} = [\beta _i - 1.96 \cdot STD\_residual, \beta _i + 1.96 \cdot STD\_residual] \end{aligned}$$

Then to assess the significance associated with different forcings estimations, we check whether the CI of different coefficients has zero. When zero is excluded from the CI, then the corresponding forcings are significant in terms of statistics in explaining HadCRUT SAT observational shifts.

### Area harvested for rice cultivation and methane emission datasets

Data on the area harvested for rice cultivation can be accessed through the Food and Agriculture Organization database (FAOSTAT) at https://www.fao.org/faostat/en/#data/QCL. Methane emission data has been sourced from https://ourworldindata.org/greenhouse-gas-emissions by^71^. This study takes methane emissions from agricultural activities.

## Supplementary Information

Supplementary Information.

## Data Availability

All the CMIP5 model datasets are available at https://esgf-data.dkrz.de/search/cmip5-dkrz/. Observational data from HadCRUT5 is available at https://crudata.uea.ac.uk/cru/data/temperature/. The area harvested for rice cultivation can be accessed through the Food and Agriculture Organization database (FAOSTAT) at https://www.fao.org/faostat/en/#data/QCL. Methane emission data has been sourced from https://ourworldindata.org/greenhouse-gas-emissions by [71].

## Code availability

The ROF method [48] used in this study is available at https://www.umr-cnrm.fr/spip.php?article23&lang=en.
