# Supplementary material for: Africa’s booming rice cultivation is fueling regional warming
Source: Sci Rep. 2025 Dec 1;15:42895. doi: 10.1038/s41598-025-27436-5 (PMC12669579; doi:10.1038/s41598-025-27436-5)
Supplement: Supplementary file 1 — Supplementary Information. [file 41598_2025_27436_MOESM1_ESM.pdf]

Africa's booming rice cultivation is fuelling regional warming:- Supplement

Basudev Swain[1*], Marco Vountas[2], Aishwarya Singh[3,4,5], Rui Song[6], Nidhi L. Anchan[7], Nisha Patel[8], Debashis Tripathy[9], Biswa R. Swain[10], Dukhishyam Mallick[11], Richard Alawode[12], and Sachin S. Gunthe[3]

[1]Atmospheric, Oceanic and Planetary Physics, University of Oxford, U.K
[2]Institute of Environmental Physics, University of Bremen, Germany
[3]Centre for Atmospheric and Climate Sciences, Indian Institute of Technology Madras, Chennai, India
[4]School of Earth and Atmospheric Sciences, Georgia Institute of Technology, Atlanta, GA, U.S.A
[5]Max Planck Institute for Chemistry, Biogeochemistry Department, Mainz, Germany
[6]National Centre for Earth Observation, Atmospheric, Oceanic and Planetary Physics, University of Oxford, UK
[7]Department of Energy, Environmental, and Chemical Engineering, Washington University, Missouri, U.S.A
[8]Department of Environmental Meteorology, University of Kassel, Kassel, Germany
[9]Yusuf Hamied Department of Chemistry, University of Cambridge, UK
[10]Chester F. Carlson Center for Imaging Science, Rochester Institute of Technology, USA
[11]IJCLab Orsay, CNRS/IN2P3, Université Paris-Saclay, Saclay, France
[12]Leipzig Institute for Meteorology, Leipzig University, Leipzig, Germany
[*]Corresponding, Basudev Swain, and Marco Vountas: vountas@iup.physik.uni-bremen.de, and basudev.swain@physics.ox.ac.uk

ABSTRACT

The significant increase of surface air temperature in Africa during the recent industrial period has been previously attributed to emissions from rapidly growing urbanization and industrial emissions. This study highlights the rapid growth of rice cultivation as another major influencing factor. We estimate that a 436% (14 million hectares) surge in rice cultivation area during the industrial period (1960-2018) in the sub-Saharan African region is associated with an increase of 603 million tons of agricultural methane emissions, making it the largest source of methane compared to other sectoral sources. These changes are further associated with an increase in the total surface air temperature anomaly to 1.3℃, with greenhouse gas (GHG) forcing alone accounting for a rise from 0.47℃ to 0.92℃ during the industrial period (1955–2005) relative to the pre-industrial baseline (1850–1900), as estimated using the Regular Optimal Fingerprinting (ROF) method. Continued rice cultivation expansion to feed Africa's rapidly growing population holds the potential for further intensifying current and future warming conditions. However, adopting more sustainable rice farming practices can help to reduce emissions and mitigate these effects.

Sl.No.	Model Name	Historical	Historical Aaer	Historical GHG	Historical NAT
1	bcc-csm1-1	Y	N	Y	Y
2	BNU-ESM	Y	N	Y	Y
3	CanESM2	Y	Y	Y	Y
4	CCSM4	Y	Y	Y	Y
5	CESM1-CAM5	Y	N	Y	N
6	CNRM-CM5	Y	N	Y	Y
7	CSIRO-MK3	Y	Y	Y	Y
8	FGOALS_g2	Y	Y	Y	Y
9	GFDL-CM3	Y	Y	Y	Y
10	GFDL-ESM2M	Y	Y	Y	Y
11	GISS-E2-H	Y	Y	Y	Y
12	GISS-E2-R	Y	Y	Y	Y
13	HadGEM2-CC	Y	N	N	N
14	HadGEM2-ES	Y	N	Y	Y
15	inmcm4	Y	N	N	N
16	IPSL-CM5A-LR	Y	Y	Y	Y
17	IPSL-CM5A-MR	Y	N	Y	Y
18	MIROC-ESM	Y	N	Y	Y
19	MIROC5	Y	N	N	N
20	MPI-ESM-LR	Y	N	N	N
21	MRI-CGCM3	Y	N	Y	Y
22	NorESM1-M	Y	Y	Y	Y
	Total	22	10	18	17

Table S1. Table containing information about different models used in this study. Our study utilizes 67 CMIP5 historical simulations. The historical simulations incorporate various forcings, with 'Y' indicating the inclusion of a particular forcing and 'N' denoting its absence. The forcings include anthropogenic aerosols (Aaer), well-mixed greenhouse gases (GHG), and natural forcings (NAT). All forcing information was sourced from the CMIP5 website: http://cmip-pcmdi.llnl.gov/index.html.)

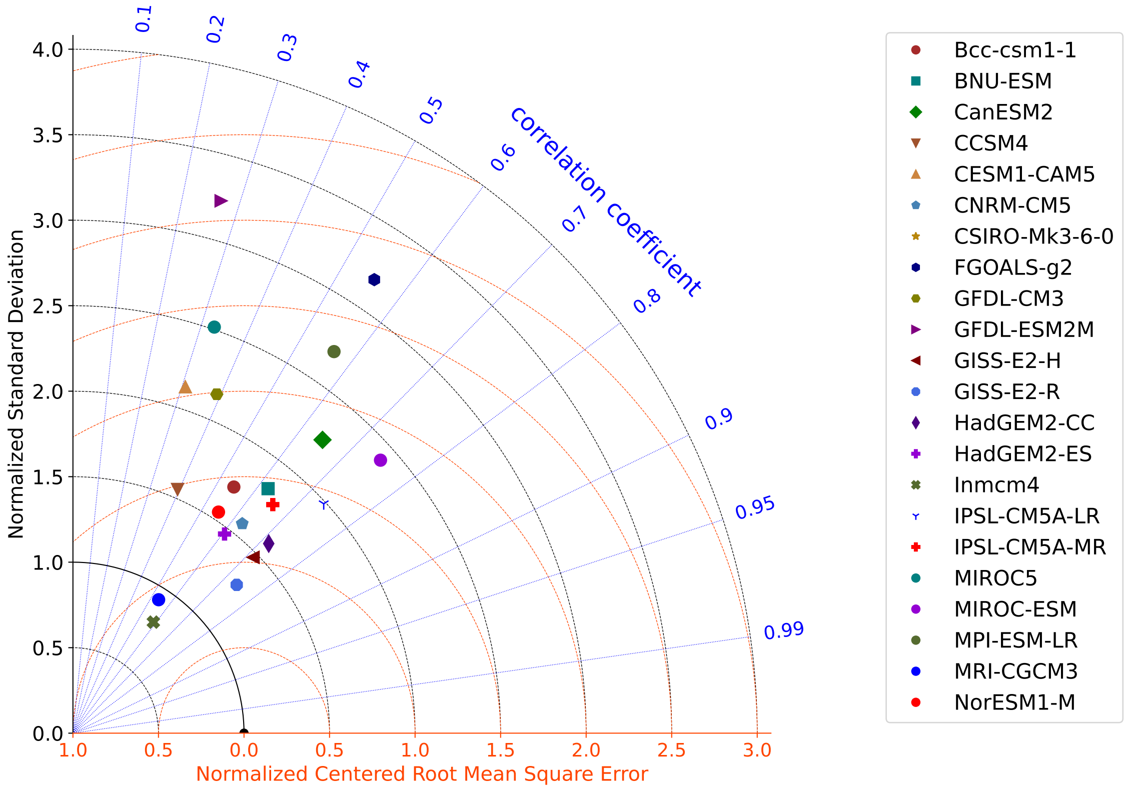

Figure S1. Taylor diagram presenting evaluation of surface air temperature simulated by different models with respect to HadCRUT5 observations. The Taylor diagram illustrates the mean surface air temperature comparison among HadCRUT5, and individual CMIP5 models from 1955 to 2005. Solid lines represent standard deviations, while dotted lines indicate correlations.

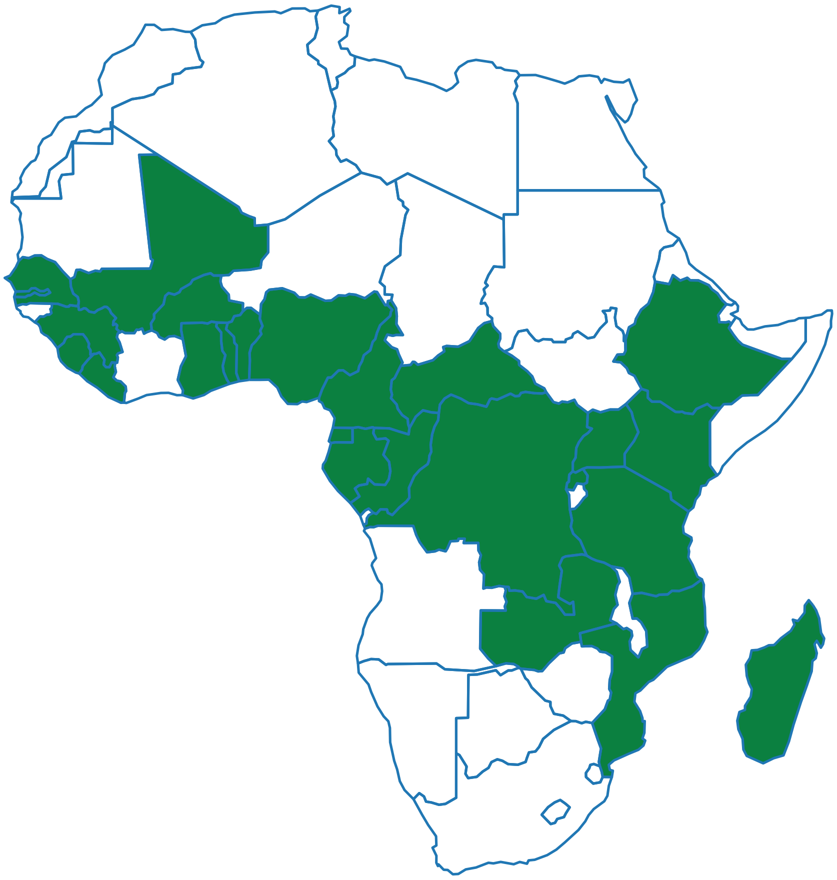

Figure S2. A map highlighting 23 Sub-Saharan countries with rapid growth in area harvested for rice cultivation. The countries are selected on the basis that the Coalition for African Rice Development (CARD) has significantly influenced rice cultivation strategies across the continent. Through its investment frameworks and national agricultural plans, CARD encourages African countries to boost agricultural productivity and to improve food security.
